# Lessons Learned: Patient Communication During the Pandemic

**DOI:** 10.5704/MOJ.2103.002

**Published:** 2021-03

**Authors:** EHM Wang, I Real, A David-Wang, DA Rubio, CL Gaston, AJ Quintos, C Dimayuga, E Dacanay

**Affiliations:** 1 Department of Orthopaedics Tumor Service, University of the Philippines Manila, Manila, Philippines; 2 Section of Medical Oncology, University of the Philippines Manila, Manila, Philippines; 3 Section of Pulmonary Medicine, University of the Philippines Manila, Manila, Philippines

**Keywords:** pandemic, tumour, patient, communication, telemedicine

## Abstract

A series of mortalities among musculoskeletal tumour patients secondary to medical illnesses during the first few months of the pandemic highlighted the need to review our methods of communication with patients. Prominent among patients’ concerns had been a fear of consulting at hospitals and a lack of ready access to health care. Recommendations are made for proactive consultation and patient education, identifying at-risk patients for follow-up and probing for possible co-morbidities. Telemedicine use is encouraged bearing in mind its inherent limitations. A network of physicians and pharmaceutical representatives is an added help we can offer our patients who may be isolated by community quarantine.

## Introduction

The first cluster of novel coronavirus infection reported in Wuhan, China has mushroomed into a global pandemic, affecting over 65 million people with over 1.5 million deaths worldwide^1^. Countries responded initially by enforcing lockdown or enhanced community quarantine (ECQ) in an attempt to contain further spread. In the Philippines, complete lockdown was enforced March 16, 2020^2^. Several tertiary government hospitals, including our center, the University of the Philippines-Philippine General Hospital, were declared COVID-hospitals by the Department of Health, severely curtailing non-COVID related admissions and surgeries in these centers. Private hospitals, overwhelmed by the unexpected initial wave of COVID patients, were also forced to redirect personnel and logistics to address the viral onslaught^3^.

In response to these government and hospital directives, surgical lists were quickly completed, prioritising emergent and urgent cases while cancelling elective surgeries and post-operative in-hospital stays were cut short in order to free up hospital beds for the anticipated deluge. Surgeries on patients not previously admitted were put on hold and cases re-evaluated for urgency of care. Even as musculoskeletal malignancies were given priority, the number of cases also saw a gradual decline after completion of the initial surgical list^4^. This was likely due to travel restrictions, fear of hospital visits with some patients opting to defer surgeries, and the cessation of face to face out-patient consultations.

## Unfortunate Events

But even as the number of orthopaedic tumour surgeries decreased, we received reports of some of our musculoskeletal cancer patients being brought to the emergency room, both at our center and in other medical facilities, only to succumb to seemingly unrelated medical conditions.

J.G. 38/M was scheduled for an above-knee-amputation for a large synovial sarcoma of the left leg. Lung metastases and primary tumour remained unchanged despite neoadjuvant chemotherapy. Just before surgery, ECQ was enforced and J.G. started on oral targeted therapy while awaiting surgery schedule. During this time, despite developing a persistent cough, he deferred consultation for several weeks due to fear of COVID infection until he became dyspneic and febrile. At the emergency room, he was intubated; a diagnosis of community-acquired pneumonia was made but he expired shortly after intubation.

P.L. 68/F had a recurrent malignant melanoma of the left upper arm for which she deferred surgery since start of the pandemic. She finally decided on surgery four months into the pandemic but while initiating clearances for surgery, she developed fever and disorientation. At the emergency room, it was discovered she had urosepsis and though she had earlier urinary tract symptoms, she had been wary to seek consultation. She succumbed to fatal arrhythmia triggered by the systemic infection.

A.M.F. 52/F had papillary thyroid CA with a large metastatic left proximal humerus lesion. In preparation for possible surgery, the tumour was subjected to arterial embolisation, shortly after which a lockdown was declared. The patient returned home to a nearby province and could not travel back to Manila for follow-up or treatment. Neither was she able to secure appropriate medication or schedule a clinic visit in her hometown. Two weeks later, she developed chills and vomiting and was brought to the emergency room of a local hospital where she expired. Cause of death was unknown to us.

What had happened? While awaiting surgeries, patients had developed other medical problems but they had either refused to consult at hospitals or were unable to access appropriate medical care. Both scenarios might be a reflection of insufficient communication between health care providers and patients; even if the former had prescribed medications, clarified surgical schedules and provided general instructions for continuing care.

## Lessons Learned

Providing instructions to patients similar to pre-COVID times may be inadequate as there are other important factors in play which impact on patient health-seeking behaviour during a pandemic. For example, it is important to recognise the increased level of distress that cancer patients and their families might face not only about their diagnosis and treatment but also about the pandemic itself^5^. Chen *et al*, in a prospective study on lung cancer patients in a Taiwanese hospital during the SARS epidemic, showed that nearly two-thirds of patients were afraid of entering a hospital for fear of acquiring SARS, and some patients ceased chemotherapy altogether^6^. This was also evident in a group of noncancer paediatric patients where fear of infection with SARS-CoV-2 prevented parents from accessing hospital care, resulting in admissions to ICU and non-COVID related mortalities^7^. Patients need to be reminded to seek continuing medical care, and equally importantly, be reassured that visits to health providers are safe and possible as long as proper precautions are undertaken. Clinicians, in general, should go beyond their normal scope of work to educate the public and allay their fear and concerns, and assure them that they still have access to high-quality, multidisciplinary care for any medical (illness)^8^.

It may also be necessary, especially during the pandemic, to provide proactive follow-up on select high-risk patients rather than rely on them to follow-up on their own. This group of patients would include those on adjuvant treatment, whether chemotherapy or radiotherapy, patients with malignant neoplasms and older aged patients.

Telemedicine is now routinely used to communicate with patients remotely via hospital hotline and expanded telehealth capabilities^3,5^. Telemedicine has been demonstrated to improve access to care and decrease health care costs^9^. But while virtual care is one of the best alternatives to reduce hospital footprint, it is also still a method of communicating with patients relatively unfamiliar to clinicians. The details and subtleties of interactions with patients, such as body language and verbal responses may be difficult to capture in teleconsultations^10^. As such, doctors may be unable to capture and patients unable to fully express their fears about both their condition and their concerns about visits to health care facilities.

It will be helpful therefore for the clinician during teleconsultation with a cancer patient to remember the latter’s vulnerability to infection and the frequent presence of accompanying comorbidities in cancer patients. Probing questions can be asked by the clinician in an attempt to ferret out these problems. Exactly how susceptible cancer patients are to infection with severe acute respiratory syndrome coronavirus 2 (SARS-CoV-2) has yet to be established. But patients with compromised immune systems are thought to be particularly vulnerable. Individuals who are undergoing active treatment with chemotherapy or radiotherapy are deemed to be at heightened risk of severe illness from COVID-19^11^. Similarly, Omarini *et al*^12^ noted a much higher rate of mortality (22%) among cancer patients on active anticancer drugs compared to the population without cancer of the same age. Restriction of movement and confinement to homes with limited walking areas in patients whose musculoskeletal cancers already limit mobility can only further adversely affect both physical and mental health as was evident in the presented patients.

At the same time, comorbidities have been consistently found to adversely affect cancer survival^13^. Our second patient had recently developed a urinary tract infection which had not been identified and managed during the ECQ. Left untreated, she developed urosepsis and eventually succumbed to the infection. The negative impact of comorbidities on cancer is amplified by their suboptimal treatment during the COVID pandemic, whether from difficulty accessing health care or from the patient’s own fears about visiting health care facilities.

Finally, as it is not always possible to predict which health facilities will remain open during a pandemic, clinicians can proactively identify a physician network and provide patients with a list of clinicians they can consult with in their hometown. The same can be done for representatives of pharmaceutical companies in order for patients to have easy access to cancer medications. Upon declaration of ECQ, patient 3 could neither travel back to our hospital nor access specialists and medications in her hometown. While these problems are true around the world, some of them are further highlighted in the setting of low and middle-income countries (LMIC)^14,15^.

## Recommendations

In summary, a series of mortalities during the start of the pandemic among musculoskeletal tumours patients awaiting surgery highlights the need for us clinicians to:

1. Maintain open lines of communication with patients at all times
2. Actively educate patients about the ongoing pandemic
3. Allay patients’ fears about hospital visits, reassuring them about hospital safety as long as everyone is properly protected
4. Proactively identify at-risk patients and regularly follow-up on them rather than wait for them to follow-up
5. Actively use telemedicine but be aware of its inherent limitations especially the difficulty of identifying the details and subtleties of interactions more readily detectable in physical consultations
6. Remember that cancer patients can be more prone to infections and have associated comorbidities which may remain undetected; thus, the need to try and ferret out these problems with probing questions
7. Proactively identify a physician and pharmaceutical network to provide healthcare and medicine access to patients who may be stranded in their hometowns due to community quarantine

While these modifications to our communication with patients are modeled and implemented during this pandemic, they may very well be adopted for future pandemics and calamities in order to further improve health care delivery to our patients.
